# On the Threshold of Illness and Emotional Isolation

**DOI:** 10.3201/eid1205.AC1205

**Published:** 2006-05

**Authors:** Polyxeni Potter

**Affiliations:** * Centers for Disease Control and Prevention, Atlanta, Georgia, USA

**Keywords:** Art and science, emerging infectious diseases, Andrew Wyeth, Christina Olson, disability, emotional isolation, tuberculosis

**Figure:**
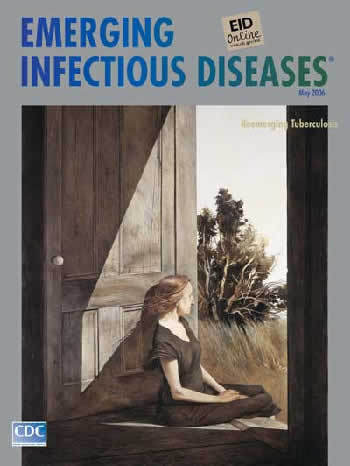
Andrew Wyeth (b. 1917). Christina Olson (1947).

"I was very thin and nervous so my father and mother took me out of school and had me tutored at home," recalls Andrew Wyeth about his education after the third grade (*1*). The ailment that kept him away from his peers during childhood and adolescence was a "sinus condition," later diagnosed as tuberculosis. "I played alone and wandered a great deal over the hills, painting watercolors that literally exploded, slapdash over my pages, and drew in pencil or pen and ink in a wild and undisciplined manner" (*2*). His precocious artistic talent was also reigned in and cultivated in the home environment. He learned from his father, painter, muralist, illustrator Newell Wyeth, who studied with foremost 19th-century illustrator, Howard Pyle (*3*).

Influenced by Henry David Thoreau and the transcendentalists, Newell Wyeth promoted awareness of the metaphorical and metaphysical value in even the most mundane objects and advocated attention to and alignment with the subtleties of the natural world. He believed that "…a man can only paint that which he knows even more than intimately…. And to do that he has got to live around it, in it and be *part* of it" (*4*). His young son spent hours painting objects, only to lament that he could "never get close enough to an object or inside of it enough" (*5*), but the telescopic views of the domestic implements he painted throughout his career show this early still-life training.

At Chadds Ford, the farming village in Pennsylvania where he was born, Wyeth worked with charcoal and oils and studied the masters, among them Albrecht Dürer, an early source of inspiration. In Maine, where the family spent summer months, he experimented with watercolors and painted landscapes in the style of Winslow Homer, with whom along with Thomas Eakins, he felt strong kinship.

"They look magnificent, and with no reservations whatsoever, they represent the very best watercolors I ever saw," wrote his father about the works in 20-year-old Andrew's first show in New York City (*6*). The critical acclaim of this and other early shows did not satisfy the young Wyeth, who thought his work too spontaneous and facile. "I was skimming along on a very superficial level. I had a terrible urge to get deeper, closer to nature" (*7*). His brother-in-law Peter Hurd, also an artist, introduced him to egg tempera, a medium that, in combination with the drybrush method he favored, slowed down his technique and added distinctive texture to the work.

"When he died," Wyeth said of his father, "I was just a clever watercolorist—lots of swish and swash…. I had always had this great motion toward the landscape, and so with his death, …the landscape took on a meaning—the quality of him" (*2*). The emotional content of the work intensified, and he began to paint figures, mostly portraits of single figures. He abstracted interfering elements, reducing the picture to an object or surrounding that captured their essence, while the figures themselves were no longer present. Trodden Weed (1951), a painting said to have been admired by Nikita Khrushchev, shows only a man's booted legs walking across the grass. This work, conceived while Wyeth recuperated from surgery to remove part of his lung, was intended as self-portrait.

Wyeth's work from Pennsylvania and Maine drew from an anti-modern, anti-urban sentiment prevalent in the United States between the Civil War and World War II (*8*). Era art favored idealized rural scenes as antidote to ongoing rapid social and economic change. Wyeth reinvented and reshaped this pictorial backdrop. Georgia O'Keefe, John Marin, and others in this period examined images and objects in their locales for universal metaphors. Edward Hopper and Charles Sheeler, among others, used common objects to depict the poor and dispossessed. To objects, Wyeth added intense personal associations, meaning, and emotion (*3*).

About abstract expressionism, a modern art movement (Piet Mondrian, Max Ernst) dominating the scene as Wyeth came of age, he remained ambivalent. "My aim is to escape from the medium with which I work. To leave no residue of technical mannerisms to stand between my expression and the observer…. Not to exhibit craft but rather to submerge it…" (*9*).

During one of his trips to Maine, Wyeth met Christina Olson and her brother Alvaro, who lived in a dilapidated, peaked-roof farmhouse in Cushing. Christina, disabled from poliomyelitis or some aggressive form of arthritis, had difficulty walking. Her strength and perseverance intrigued and inspired him, and during their long friendship, he kept a studio in the Olson household. Christina's World (1948), the image, from the back, of his friend in a large field crawling toward her home, became one of the most recognized American paintings. "I felt the loneliness of that figure—perhaps the same that I felt myself as a kid," Wyeth said of the work (*2*).

Christina Olson, on this month's cover, allows a more generous glimpse of the figure's profile of illness, disability, and their psychological outcome. In Wyeth's words, "a wounded gull," Christina has been left behind. Alone, she rests on the threshold, her body propped against the open door, hair blown softly in the breeze. Her posture in the center of the painting, erect and dignified, defies the somber aspect of her wasting limbs. Unable to join in, she seeks a lighted spot, an outlet into normalcy. And, "in the moment," she soaks up the sun, connecting with the universe.

Wyeth's empathetic portrait of his friend's physical impairment symbolizes the limits imposed by illness, in her case, undiagnosed and misunderstood, in his, finally named tuberculosis. Current efforts, whether skin testing of children at risk (*10*) or genotyping of *Mycobacterium tuberculosis* strains (*11*), lessen the life-defining impact of this disease and lower the threshold of illness and emotional isolation.
